# Optimizing HIV/AIDS resources in Armenia: increasing ART investment and examining HIV programmes for seasonal migrant labourers

**DOI:** 10.7448/IAS.19.1.20772

**Published:** 2016-06-07

**Authors:** Sherrie L Kelly, Andrew J Shattock, Cliff C Kerr, Robyn M Stuart, Arshak Papoyan, Trdat Grigoryan, Ruben Hovhannisyan, Samvel Grigoryan, Clemens Benedikt, David P Wilson

**Affiliations:** 1 The Kirby Institute, UNSW, Sydney, Australia; 2 National Center for AIDS Prevention, Yerevan, Armenia; 3 The World Bank, Washington, DC, USA

**Keywords:** HIV, HIV investment, people living with HIV, HIV treatment, seasonal labour migrant HIV programmes

## Abstract

**Introduction:**

HIV prevalence is declining in key populations in Armenia including in people who inject drugs (PWID), men who have sex with men, prison inmates, and female sex workers (FSWs); however, prevalence is increasing among Armenians who seasonally migrate to work in countries with higher HIV prevalence, primarily to the Russian Federation.

**Methods:**

We conducted a modelling study using the Optima model to assess the optimal resource allocation to meet targets from the 2013 to 2016 national strategic plan to minimize HIV incidence and AIDS-related deaths by 2020. Demographic, epidemiological, behavioural, and programme cost data from 2000 through 2014 were used to inform the model. The levels of coverage that could be attained among targeted populations with different investments, as well as their expected outcomes, were determined. In the absence of evidence of the efficacy of HIV programmes targeted at seasonal labour migrants, we conducted a sensitivity analysis to determine the cost-effective funding threshold for the seasonal labour migrant programme.

**Results:**

The optimization analysis revealed that shifts in funding allocations could further minimize incidence and deaths by 2020 within the available resource envelope. The largest emphasis should be on antiretroviral therapy (ART), with the optimal investment to increase treatment coverage by 40%. Optimal investments also involve increases in opiate substitution therapy and FSW programmes, as well as maintenance of other prevention programmes for PWID and prevention of mother-to-child transmission. Additional funding for these increases should come from budgets for general population programmes. This is projected to avert 17% of new infections and 29% of AIDS-related deaths by 2020 compared to a baseline scenario of maintaining 2013 spending. Our sensitivity analysis demonstrated that, at current spending, coverage of annual testing among migrants of at least 43% should be achieved to warrant continuation of funding for this programme.

**Conclusions:**

Optimization of HIV/AIDS investment in Armenia, with a main priority for scaling-up ART, and less emphasis on primary prevention in the general non-key population could significantly reduce incidence and deaths by 2020.

## Introduction

Levels of HIV funding from international donors to lower-middle income countries, like Armenia, have plateaued [1]. With an increased need to respond to the HIV epidemic in Armenia, but with international resources becoming more limited, it is more essential than ever before to ensure that programmes can do more with less by focusing the HIV response and strategically targeting resources. As such, to meet the HIV response targets outlined in the 2013 to 2016 national strategic plan for the Republic of Armenia [2], the Government of Armenia are continuing to develop their HIV allocative efficiency investment case. Allocative efficiency refers to the process of targeting available resources towards the most cost-effective mix of interventions to achieve optimal health outcomes [3]. Optima, a mathematical model of HIV transmission and disease progression, was used to conduct this allocative efficiency analysis.

The Republic of Armenia is an eastern European country with a population of 3 million people. While it has a relatively low estimated prevalence of HIV among people aged 15 to 49 years, 0.19% in 2013 [4], this prevalence has been gradually increasing from 0.12% in 2006. Since 1988, there have been 2051 registered cases of HIV, with 334 new cases registered in 2014 alone [5]. Currently, HIV is mainly being transmitted by heterosexual exposure. Heterosexual transmission has increased by over twofold from 30.6% in 2004 to 73.9% in 2013 [6]. Conversely, transmission among people who inject drugs (PWID) has declined by fivefold from 67.3% to 13.4% in the same period.

With increasing transmission via heterosexual interactions, key populations in Armenia most-at-risk for this type of transmission are female sex workers (FSWs) and their clients, and seasonal migrant labourers. Since HIV prevalence is declining in FSWs and their clients, but increasing in seasonal migrant labourers, studies have been conducted to examine the status and cause of HIV transmission in this group [7,8]. Findings show that as a result of poor socio-economic conditions in the country an estimated 70,000 Armenians migrate outside the country each year seeking seasonal labour, an estimated 93% migrate to the Russian Federation [9]. In 2014, Russia had the highest incidence of HIV in the Eastern Europe and Central Asian region, with a prevalence of HIV much higher than in Armenia [10,11]. In 2012, 62% of the 228 people registered as being newly infected with HIV in Armenia were determined to have been infected abroad (141 cases); 126 of these cases were infected in the Russian Federation (89.4%) mainly from heterosexual transmission [8]. In turn, 20% of sexual partners of these newly infected seasonal migrant labourers became infected (45 cases). This represents 82% of the total number of cases reported in 2012 having been associated with seasonal labour migration. Similar findings have been reported since 2009 and have also been observed in other countries targeting seasonal labour migrants as part of their HIV response [12–15]. This evidence shows that migrants are especially vulnerable to becoming infected with and transmitting HIV and are of particular interest within the national strategic plan for HIV response in Armenia [2].

It is well recognized that ART prevents AIDS-related morbidity and mortality and that it can significantly reduce the risk of HIV transmission. Therefore, it follows that there is a need to extend coverage gains in all settings as part of any national strategic plan [4], and the same holds true in Armenia as our results will show.

## Methods

To represent the HIV epidemic in Armenia, we used Optima, a dynamic population-based HIV transmission and disease progression model for optimally allocating HIV resources. It uses a linked system of ordinary difference equations to track the movement of people living with HIV across five clinical categories (susceptible, undiagnosed, diagnosed, on ART with detectable virus, or on ART with suppressed virus) and five CD4 count health states as described in detail by Kerr *et al*. [3]. The overall population was divided into 12 groups by dominant risk type (FSWs, clients of FSWs, men who have sex with men (MSM), PWID, seasonal migrant labourers, and prison inmates) or general population age and sex group (females 0–14 years, males 0–14 years, females 15–49 years, males 15–49 years, females 50 years and older, and males 50 years and older). Demographic, behavioural, and biological data by population group from 2000 to 2014 [16–21] were used to inform the national Armenian epidemic model. These data were collected by in-country stakeholders as part of an allocative efficiency workshop that was held in Yerevan, Armenia, in November 2014, cohosted by the World Bank Group, UNAIDS (Joint United Nations Programme on HIV/AIDS), UNDP (United Nations Development Programme), the Global Fund to Fight AIDS, Tuberculosis and Malaria, and various other partners. The model was then aligned to the epidemiological and treatment data through a calibration process (see Supplementary files for more details).

We assessed the epidemiological impact of eight HIV programmes: ART, prevention of mother-to-child transmission (PMTCT), FSW testing and prevention, MSM testing and prevention, PWID testing, prevention, and needle-syringe programmes, opiate substitution therapy (OST), prisoner testing and prevention, and seasonal labour migrant testing and prevention on the HIV epidemic in Armenia. To model the impact of varying investment in such prevention and treatment programmes on the national epidemic, a set of spending versus transmission-related outcome relationships was developed. We refer to these potentially non-linear relationships as cost-coverage curves as illustrated in the Supplementary file (Supplementary Figure 2). Economic and coverage data [6] from the past implementation period were used to inform the shape and level of saturation of these cost-coverage curves for each programme and corresponding affected population, and type of sexual partner relationship (regular, casual, or commercial) where applicable. Non-linearity is more pronounced when additional initial investment is required to implement a programme compared with programmes which can be operational quickly with little resources. The maximum reach of a programme affects its non-linearity for relatively large amounts of spending. ART programme costs are driven by the unit cost of antiretroviral drugs, with cost-coverage curves assumed to be linear with no scaling effect. To note, the ART programme does not include HIV testing and counselling (HTC), this is represented in a separate programme with a separate budget item to ART. HTC coverage represents the percentage of the target population who were tested and know their HIV status for a given amount of spending. For some cost-coverage curves, at zero-spending a certain level of outcome can be expected. For example, without spending on condom distribution programmes from the HIV budget, a certain proportion of the population will still use condoms.

The model employs an optimization algorithm, described elsewhere [22], which uses the cost-coverage curves to assess the impact of different resources allocations on the national HIV epidemic. This algorithm then efficiently determines the optimal distribution of HIV investment to minimize new HIV infections and AIDS-related deaths. An optimization using the total 2013 HIV budget of US$3,907,959 was generated to best achieve the national strategic targets in Armenia to minimize infections and deaths from 2015 through 2020. Spending allocation uncertainty bounds for each programme for this optimization were generated (Supplementary Figure 4). Management and other indirect costs were considered to be “fixed” costs in this study and were not considered as part of the optimization. Lastly, as of 2015 service delivery for HTC programmes for the general population was to be covered under key population programmes; therefore, this programme was no longer a stand-alone programme and was therefore not considered in the optimization.

Using the Optima model, we also varied the 2013 HIV budget amount from 0% to 200% in 20% increments and optimized the allocation from 2015 through 2020 to minimize cumulative HIV incidence and AIDS-related deaths using the eight HIV programme listed compared to the actual 2013 HIV programme allocation. This analysis was performed to determine prioritization of programme funding and to provide guidance to the Government of Armenia if more or less funding were to become available in the future.

A main focus of the Armenian Government is to prevent HIV transmission and increase testing and counselling coverage in seasonal labour migrants. This has a twofold benefit: (1) primarily, if found to be HIV-positive, then migrant workers can initiate ART and also take other precautions to prevent onward transmission to their female partners in Armenia; (2) education may potentially lead to awareness which may reduce their risk of acquiring HIV while working abroad. Since HIV programmes targeted at seasonal labour migrants in Armenia were only implemented in 2013, the efficacy of these programmes has yet to be determined; therefore, we examined outcomes required for these programmes to warrant increased funding within the framework of the optimized HIV budget. To accomplish this, we conducted a sensitivity analysis of the seasonal labour migrant HTC programme to determine the cost-effectiveness threshold within the optimization for this programme. We focused on the HTC component of this programme as it was determined to have the greatest impact on the optimization compared to the condom programme component. We used the baseline cost-outcome curve for the seasonal labour migrant HTC programme with current spending for this programme of US$200,707 and incrementally varied the cost-coverage curve saturation levels to generate a sensitivity curve (Supplementary Figure 3).

The Optima HIV model has been or is currently being applied in over 50 countries around the world. Notably, an analysis in Sudan revealed that optimization allowed for more funding to be allocated to key HIV programmes despite a lower total budget [23]. Compared with other commonly used HIV models, namely Goals (Spectrum) [24] and AEM (AIDS Epidemic Model) [25], Optima provides the capability to optimize HIV resource allocation.

## Results

**Figure 1:**
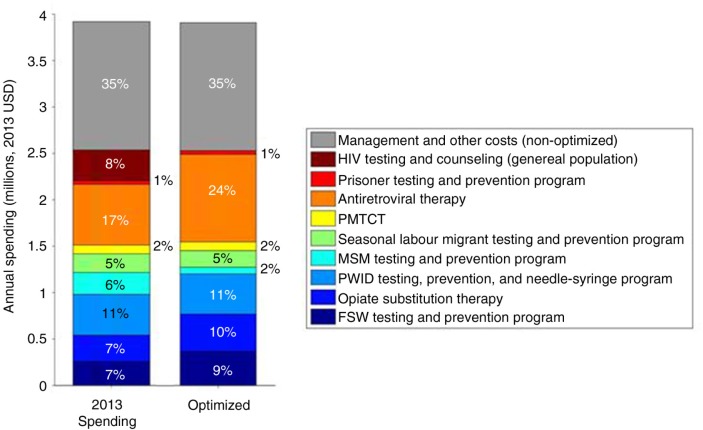
Comparison of HIV spending in 2013 in Armenia versus model-estimated optimized spending to minimize cumulative HIV incidence and AIDS-related deaths from 2015 to 2020.

To reduce new HIV infections and AIDS-related deaths in Armenia, the optimal funding allocation across HIV programmes was determined (Figure 1). We found that, as a top priority, antiretroviral therapy funding should be substantially increased from 17% of the 2013 budget (representing US$653,000 spent) to 24% (US$943,000) in 2015 and maintained to 2020, representing a 40% increase. With this increased spending, treatment coverage is projected to increase from 65% to 86% coverage of ART for people living with HIV who are eligible for treatment, that is, those with CD4 counts less than 500 cells/mm^3^.

Funding for OST should also be increased as a high priority from 7% of the total budget in 2013 (representing US$283,000 spent) to 10% (US$402,000) in 2015 and maintained through 2020. This would result in an OST coverage increase from 4% to 5%. Although HIV prevalence in PWID is declining in Armenia, OST was found to be a cost-effective programme for this optimization, and therefore, increasing investment to this programme would result in increased coverage and benefit to PWID.

Our Optima allocative efficiency analysis suggests that funding for the FSW testing and prevention programme should also be increased from 7% of the 2013 budget (representing US$256,000 spent) to 9% (US$366,000) in 2015 and maintained through 2020, corresponding to a coverage increase from 42% to 56%. Funding for testing and prevention for PWID and needle-syringe programmes, PMTCT, and prisoner programmes is to be maintained at current levels. To streamline the overall HIV programme in Armenia, HTC programmes for the general population are being incorporated as part of HTC programmes for key populations. The optimization identified the MSM programmes as being the least cost-effective programme for key populations at current budget levels, and with very limited flexible budget for the optimization, MSM programme would be the first more essential programme to have reduced funding in place of scaling-up of ART, OST, and FSW programmes; however, other considerations may indicate that some level of funding should be allocated.

**Figure 2:**
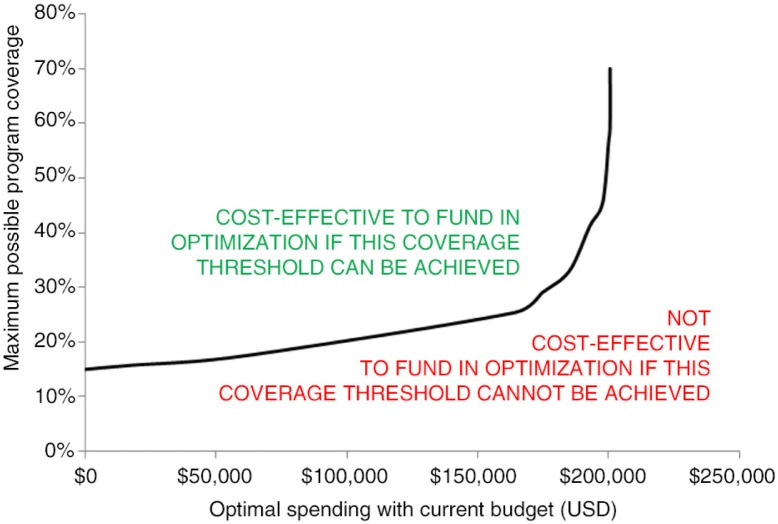
Cost-coverage sensitivity analysis for seasonal labour migrant HIV testing and counselling programmes.

We determined that it will be cost-effective to fund seasonal labour migrant programmes if a coverage threshold level for HTC for seasonal migrant labourers is at least 43% at current spending levels based on current cost-effectiveness, as illustrated in the sensitivity analysis whereby the trajectory of the curve changes from a sigmoidal to an exponential curve as shown in Figure 2. In 2013, an introductory version of the HIV migrant programme was implemented and achieved 3% coverage of migrants. Therefore, with current cost-effectiveness, if threshold coverage levels of 43% cannot be achieved by the end of 2016, it is recommended that investment to migrant programmes be reduced in 2017 and additional funding that was allocated to this programme be transferred to other programmes including increasing ART, FSW, OST, and PWID testing and prevention and needle-exchange programmes.

**Figure 3:**
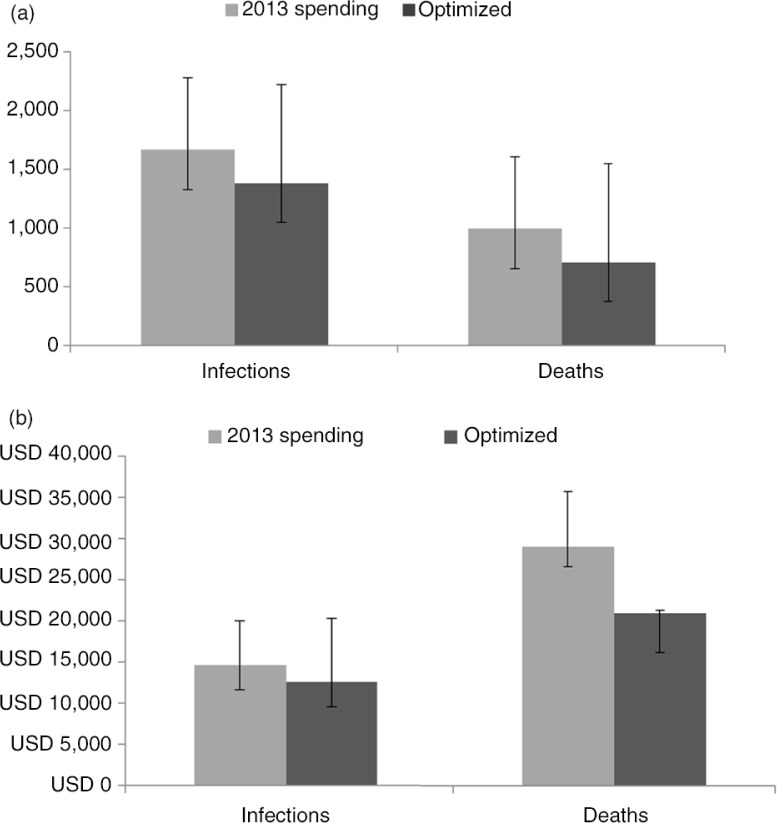
(a) Cumulative new HIV infections and AIDS-related deaths (2015–2020) and (b) cost per new infection or AIDS-related death averted (2015–2020).

Assuming that HIV programmes targeted at seasonal migrant labourers achieve the minimum threshold coverage levels for cost-effectiveness within this optimization and that this programme would be funded at optimal levels through 2020, it is projected that by optimizing allocation of HIV resources as described herein, approximately 300 new infections (17%) and 300 AIDS-related deaths (29%) could be averted between 2015 and 2020 compared to continuing to apply 2013 HIV spending patterns through the same time period (Figure 3a). This is meaningful in Armenia as the number of infections averted is approximately the same number new HIV cases that were reported in 2014 alone (334) and the cumulative number of AIDS-related deaths (443) reported since the beginning of the epidemic in Armenia [5]. In addition, it is estimated that the costs per new infection and AIDS-related death averted would be decreased in the optimized scenario. When optimizing to minimize both HIV incidence and AIDS-related deaths from 2015 to 2020, the estimated cost per new infection averted would be US$12,652 applying the same 2013 spending amount, compared to a cost per new infection averted of US$14,709 if the 2013 HIV programme allocation were maintained during the same period, representing a savings of US$2,057 per infection averted or a 16% reduction in costs (Figure 3b). Similarly, the estimated cost per AIDS-related death averted would be US$20,079 if resource allocation were optimized to minimize new infections and AIDS-related deaths from 2015 to the end of 2020, compared to costing US$8,086 or 39% more per AIDS-related death averted (US$29,079) if the 2013 HIV resource allocation were maintained for the same period.

**Figure 4:**
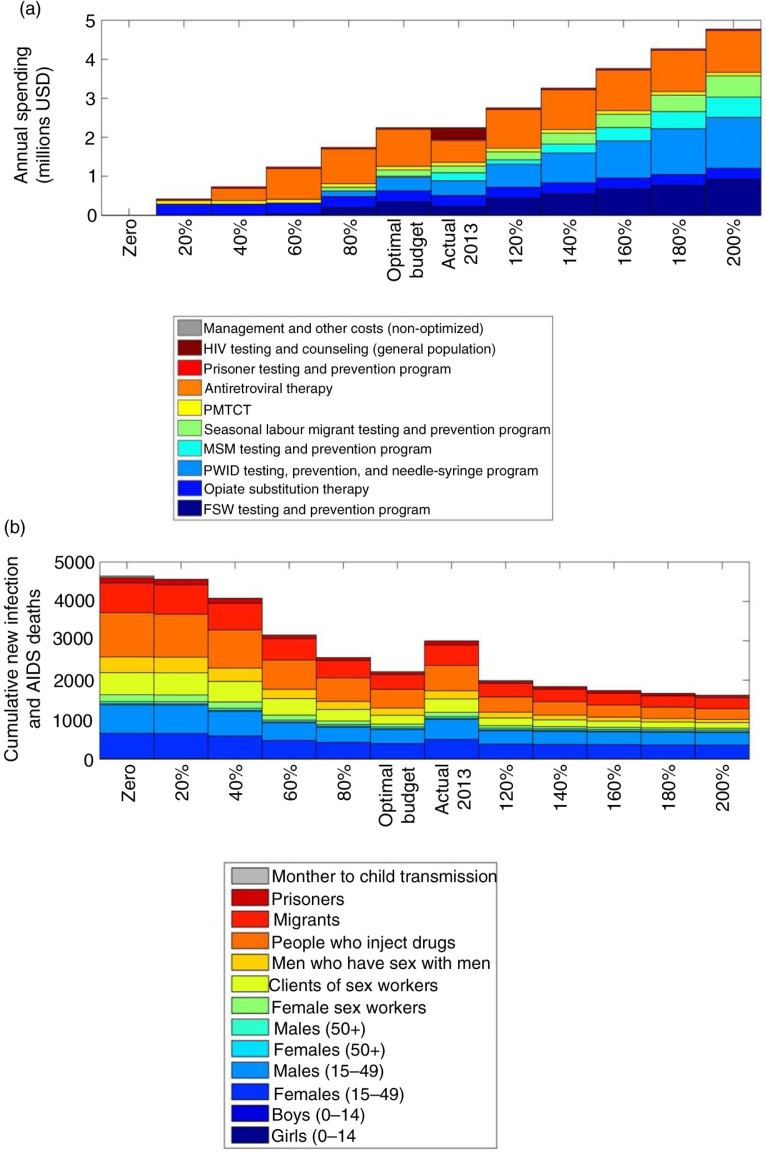
Spending allocations for varying budgets (0–200%) (a) allocated by HIV programme and (b) to minimize cumulative HIV incidence and AIDS-related deaths by 2020 specified by population group.

By varying the optimized HIV budget from 0% to 200%, we were able to identify funding to programmes that were the most impactful for minimizing new infections and AIDS-related deaths and should receive priority funding. We found that if only 80% of the 2013 budget were available from 2015 through 2020, the MSM testing and prevention programme would not be funded. If only 60% of the budget were available for the same period, the seasonal labour migrant testing and prevention and the PWID testing, prevention and needle-syringe programmes would not be funded. As well, we illustrated that the cumulative number of new HIV infections and AIDS-related deaths by population group projected by 2020 decreased markedly as the budget was increased from 20% to 200% of the 2013 HIV programme spending (Figure 4). The positive impact of minimizing new infections and AIDS-related deaths decreased more gradually as the budget was increased from 100% to 200% compared to increasing the budget from 0% to 100%.

## Discussion

As there is an increasing need to respond to the HIV epidemic in Armenia, but with almost 80% of HIV funding coming from international sources anticipated to be decreased in future and no anticipated fiscal space for increasing government spending on HIV, we conducted an allocative efficiency analysis to determine the optimal mix of HIV programme funding to minimize infections and AIDS-related deaths. Using the Optima model, we optimized the annual distribution of the 2013 HIV budget for 2015 through 2020 and showed that funding for ART and OST programmes should be increased by over 40%, and by almost 30% for FSW programmes. In addition, it is recommended that a technical efficiency analysis be conducted to explore if higher coverage can be achieved with a lower unit cost, in particular for an MSM programme, which was determined to be the least cost-effective programme for key populations in this context. An informal benchmarking analysis comparing unit costs for MSM programmes with five other countries in Eastern Europe and Central Asia showed that MSM programmes could be delivered more cost-effectively in three of the five countries. Current funding levels for testing and prevention for PWID and needle-syringe programmes, PMTCT, and prisoner programmes are to be maintained.

This optimization of HIV resources is projected to result in almost 300 fewer infections and 300 fewer AIDS-related deaths by 2020. In addition, costs per new infection or AIDS-related death averted would be reduced if spending were optimized and would lead to significant savings of 16% per new infection averted (US$2,057) and 39% per AIDS-related death averted (US$8,086). It is important to keep in mind that there is uncertainty surrounding these findings due to the lack of reliable cost-coverage data over time used to inform this analysis. Improved cost-coverage data collection will only strengthen these types of modelling exercises.

Seasonal migrant labourers are the only group for which HIV prevalence is increasing in Armenia (Supplementary Figure 1); therefore, it is crucial to ensure that programmes targeted at this group, which were only implemented in 2013, are effective. The sensitivity analysis we performed will allow the Armenian Government to evaluate over short periods of time whether HIV programmes for seasonal migrant labourers are effective and whether funding should be increased or decreased. Our analysis showed that warranting funding increases to these programmes must be supported by coverage increases of over 40% with current spending amounts to meet the cost-effectiveness threshold. As HIV prevalence has stabilized or is declining in all key populations in Armenia, other than in seasonal labour migrants, we did not conduct a sensitivity analysis for all other corresponding HIV programmes and target populations.

It will be crucial to monitor and report the effectiveness of programmes targeted at seasonal labour migrant populations in Armenia and adjust the optimized HIV spending accordingly. The vast majority of Armenian seasonal labourers migrate to the Russian Federation in search of employment and while the Russian government is obligated to provide free medical care to all its citizens, migrants are very unlikely to receive care, including HIV testing and treatment, as registration is a requirement for receiving these free health services [26] and migrant workers are typically unregistered.

As of 2013, national HIV/AIDS services have been targeted at seasonal migrants returning to Armenia. In addition, organizations like World Vision are operational in Armenia and focus on reducing the vulnerability of seasonal labour migrants returning to Armenia to HIV and STIs infection and strengthening human rights for this population group [9]. In 2004, the United Methodist Committee on Relief (UMCOR)-Armenia implemented the *Prevention and Control of HIV/AIDS, STIs and TB project* targeted at male seasonal migrants. By 2013, 154 rural Armenian communities were reached through services afforded by this project including HIV health and gender training, as well as services delivered through a mobile medical team who provided HIV testing (15% testing coverage of the 588 male migrants who participated) and counselling, condom distribution, and linkage to care [8]. These organizations are working to involve community representatives in systems and processes to support the national HIV response.

In other settings such as in Hamburg, Germany, and Rabat, Morocco, organizations are working to improve HIV outreach services to migrants [12]. In Nepal, seasonal labourers who migrate to India accounted for 46% of the estimated HIV cases reported in 2005 [13]. A study conducted in a northwest district of Ethiopia reported that seasonal labourers commonly exhibit risky sexual behaviours, which is likely to increase their susceptibility of becoming infected with HIV [14]. It has been shown that there is a complex linkage between migration, commercial sex activities, and infectious disease transmission [15]. To compound the increased risk of a migrant becoming infected with HIV, it was recently reported in *Nature* that “those most likely to be infected (by HIV), such as people who migrate to find work, are least likely to be reached by testing campaigns [27].” Upon returning to their home country, in this case to Armenia, migrant workers who have become infected with HIV abroad, but who are unaware of their HIV status, in turn put their regular sexual partners at increased risk of infection. Therefore, once the HIV testing and treatment programmes targeted at migrants are fully implemented, it will be important to monitor these programmes to assess and report their effectiveness, such that programme planning and spending can be adjusted accordingly.

Should Armenia be able to purchase antiretroviral drugs at reduced costs, these savings could be incorporated into subsequent optimization analysis. Armenia does not anticipate any new HIV technologies in the coming years which would affect these optimization results. Should new technologies or approaches be implemented, for example, PrEP (pre-exposure prophylaxis), these could certainly be incorporated in the optimization analysis.

HIV resources invested on indirect programmes to sustain an enabling environment for prevention programmes, strengthen HIV programme management and administration, as well as on social protection and social services in Armenia account for 35% of total HIV spending. In any context, it is important to examine and wherever reasonable strive to reduce indirect costs through improved technical and administrative efficiency to free up additional budget to fund direct HIV testing and treatment programmes. Any savings uncovered from efficiency gains in programme costs could be included as part of the flexible optimization budget and be applied to increase coverage of direct programmes like HIV testing and treatment. The Armenian Ministry of Health have also incorporated results from this allocative efficiency optimization analysis into their Global Fund HIV Concept Note funding application; these results have informed the future national strategic plan to shift HIV resources as described herein.

## Supplementary Material

Optimizing HIV/AIDS resources in Armenia: increasing ART investment and examining HIV programmes for seasonal migrant labourers
