# Supplementary material for: Optimizing HIV/AIDS resources in Armenia: increasing ART investment and examining HIV programmes for seasonal migrant labourers
Source: J Int AIDS Soc. 2016 Jun 7;19(1):20772. doi: 10.7448/IAS.19.1.20772 (PMC4899532; doi:10.7448/IAS.19.1.20772)
Supplement: Optimizing HIV/AIDS resources in Armenia: increasing ART investment and examining HIV programmes for seasonal migrant labourers [file JIAS-19-20772-s001.pdf]

SUPPLEMENTARY MATERIAL

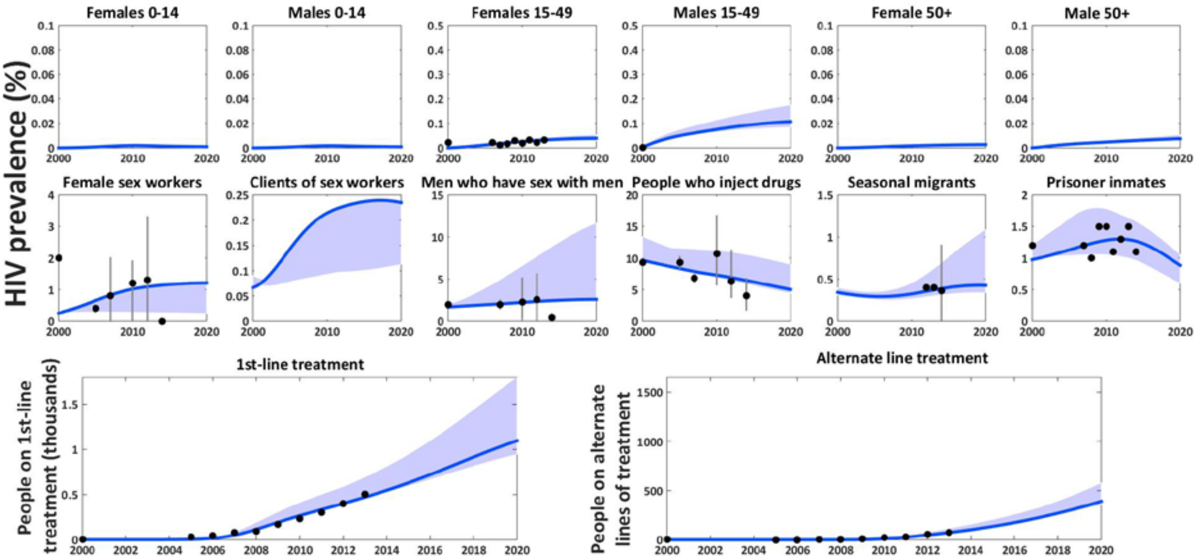

Figure S1: Modelled HIV prevalence by population group and number of people on first-line and subsequent treatment from 2000 to 2020 with uncertainty (blue shaded area)

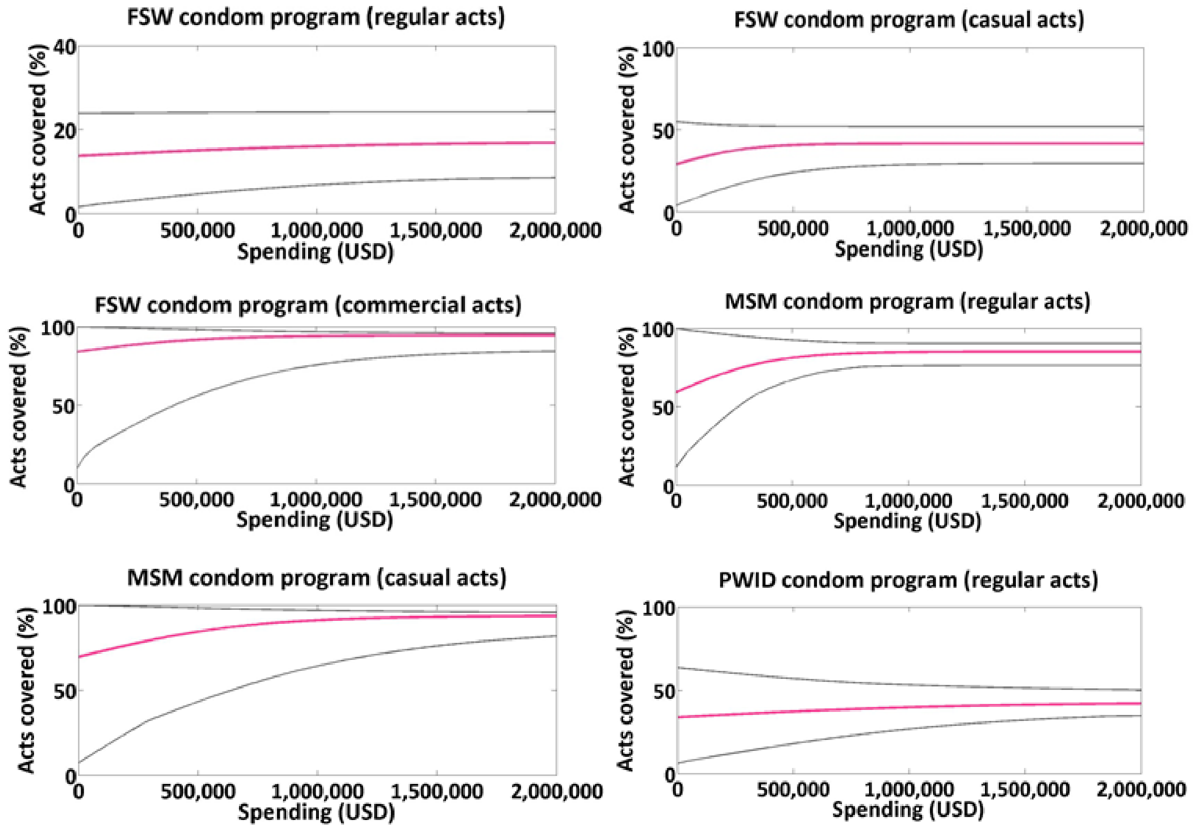

Figure S2: Cost-outcome curves by program and population group (pink curve) with uncertainty (grey curves)

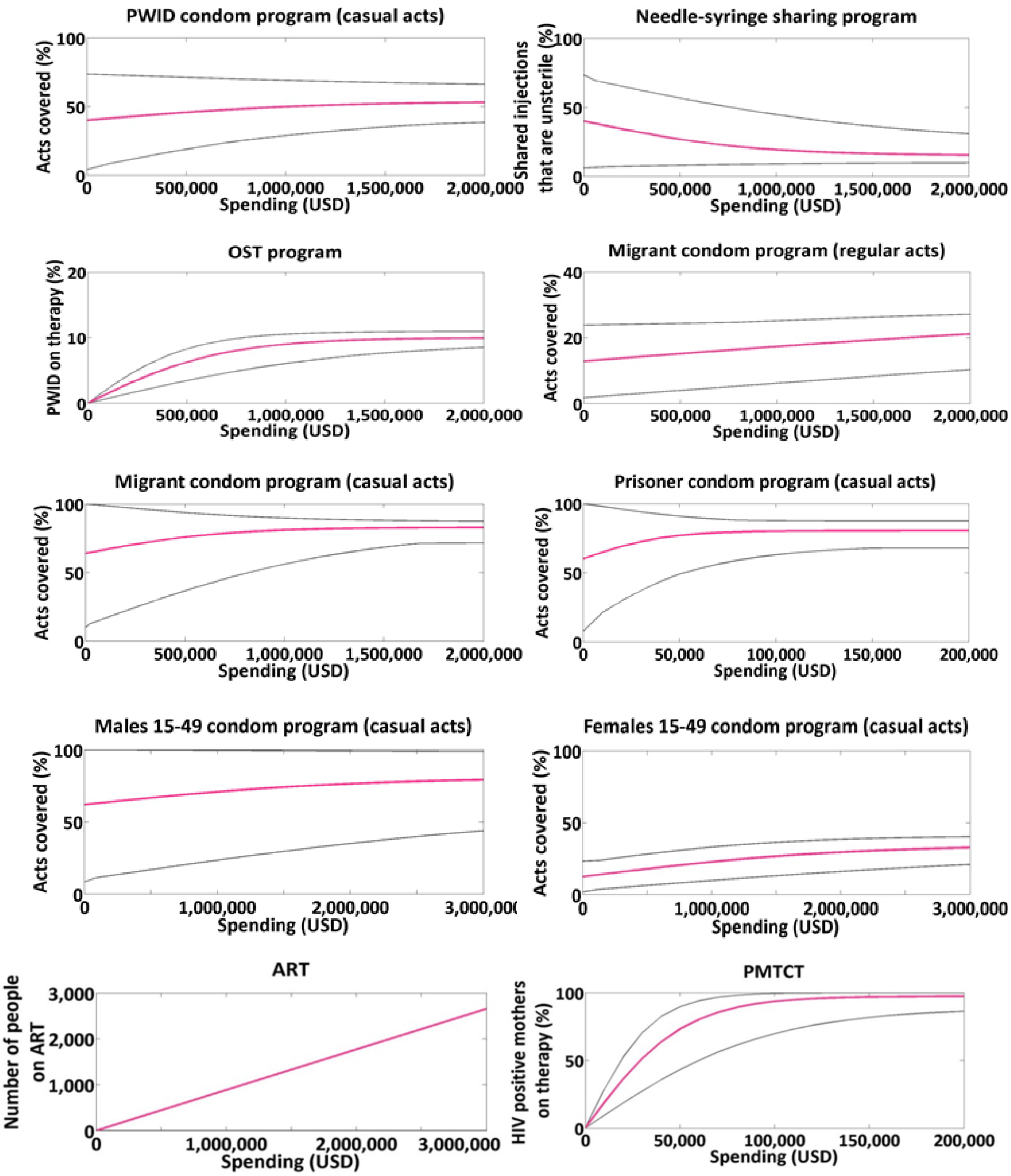

Figure S2. Cost-outcome curves by program and population group (pink curve) with uncertainty (grey curves) (continued)

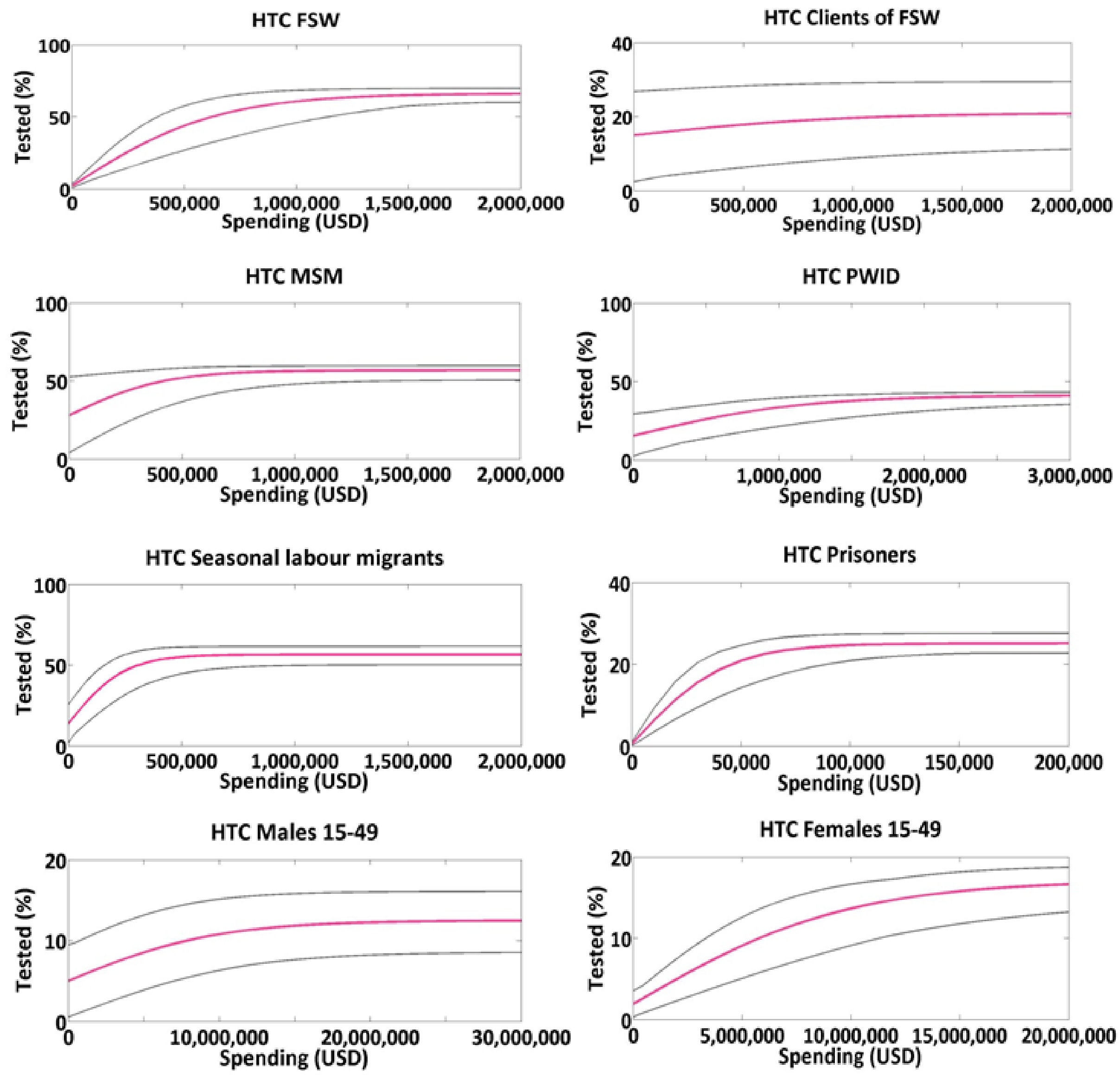

Figure S2. Cost-outcome curves by program and population group (pink curve) with uncertainty (grey curves) (continued)

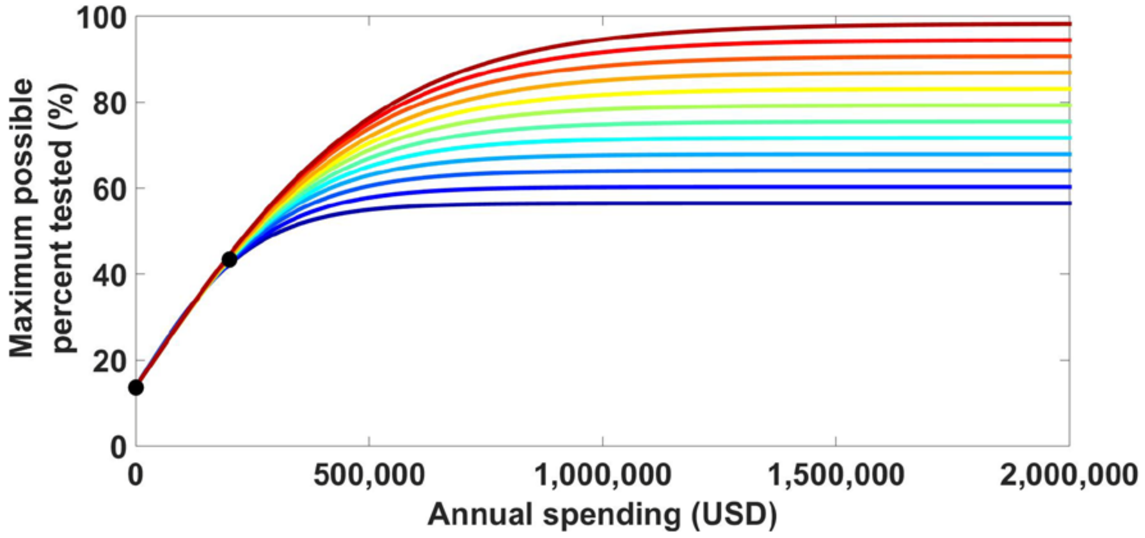

Figure S3: Variation of coverage curves for HIV testing and counselling for seasonal labor migrants for generating sensitivity analysis results

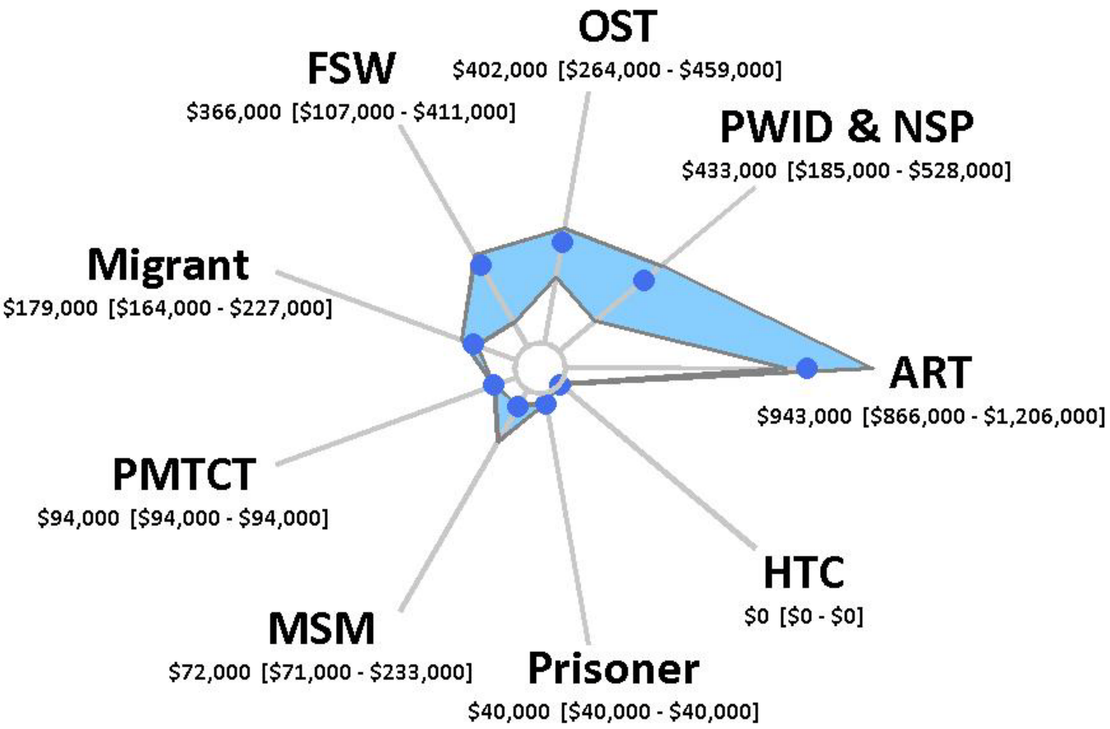

Figure S4: Uncertainty bounds for HIV program spending (USD) optimized to minimize cumulative HIV incidence and AIDS-related deaths by 2020
